# The comparison of multimodal imaging findings of central serous chorioretinopathy patients in regard to the early anatomically treatment response to half-fluence photodynamic therapy: a retrospective case–control study

**DOI:** 10.1186/s40942-017-0073-z

**Published:** 2017-06-12

**Authors:** Abdullah Ozkaya, Ruveyde Garip, Zeynep Alkin, Muhittin Taskapili

**Affiliations:** Beyoglu Eye Training and Research Hospital, Bereketzade Cami Sok., Beyoglu, 34421 Istanbul, Turkey

**Keywords:** Central serous chorioretinopathy, Fluorescein angiography, Indocyanine green angiography, Optical coherence tomography, Photodynamic therapy

## Abstract

**Background:**

To compare the multimodal imaging findings of chronic central serous chorioretinopathy (CSC) patients who are good or poor responders to low-fluence photodynamic therapy (PDT).

**Methods:**

Retrospective, interventional comparative study. The CSC patients who were admitted to our clinic for the first time between January 2013 and December 2015 were included in the study. Patients were treated with PDT only if they did not show any sign of resolution after at least 6 months from the initial signs of the disease. The patients who showed full or partial response to PDT after 3 months of treatment were accepted as good responders, those who did not show any sign of resolution were accepted as poor responders. The optical coherence tomography (OCT), fluorescein angiography (FA), and indocyanine green angiography (ICGA) findings were compared between the two groups.

**Results:**

A total of 101 eyes of 101 patients were included: 76 eyes (75.2%) were considered as good responders and 25 eyes (24.8%) as poor responders. In regards to OCT and FA findings there was not a significant difference between the two groups for all of the evaluated findings (p > 0.05 for all). In regards to ICGA findings, there was a statistically difference in the percentage of intense midphase hypercyanescence (p < 0.0001).

**Conclusions:**

The multimodal imaging findings of CSC patients were compared in regard to their PDT response. The presence of midphase hypercyanescence in ICGA seemed to be positive predictive factor for the PDT response in CSC patients.

## Background

Central serous chorioretinopathy (CSC) is a common macular disease, which is characterized by serous retinal detachment [1, 2]. Two theories have been proposed regarding the pathophysiology of CSC which are based either on choroidal dysfunction or retinal pigment epithelium (RPE) dysfunction, respectively. Although RPE dysfunction theory has yielded more attention for a long time, choroidal thickness theory has become more important after the results of indocyanine green angiography (ICGA) and enhanced depth imaging optical coherence tomography (EDI-OCT) studies [3–5]. In CSC patients, choroidal vascular anomalies and leakage was shown via ICGA, and subfoveal choroidal thickness was found to be thicker via EDI-OCT, two essential findings supporting further the role of choroidal dysfunction in the pathogenesis [3–5]. On the other hand, taking into account the important role that a normal functioning RPE plays by quickly absorbing the subretinal fluid (SRF), it is not possible to completely exclude the RPE theory from the pathogenesis. Besides, several RPE abnormalities have been described in CSC patients [6].

Central serous chorioretinopathy was a usually self-limited condition which in general spontaneously resolves within 4–6 months; therefore observation was preferred over treatment in the first months. If the serous retinal detachment persisted after the first 3–6 months or recurrence was detected after the resolution of the first episode, treatment options such as laser photocoagulation and photodynamic therapy have to be considered [7]. Photodynamic therapy was first introduced for the treatment of neovascular age-related macular degeneration (nAMD) where it was no longer used after the introduction of the anti-vascular endothelial growth factor agents [8]. However, photodynamic therapy (PDT) has been found to be an effective treatment option in polypoidal choroidal vasculopathy (PCV) and CSC which have been classified under the pachychoroid spectrum disorders [9]. It is well known that PDT targets primarily the choroid and both the choroidal leakage and thickness were shown to be decreased after this treatment in patients with PCV and CSC [5, 10]. Although not approved by Food and Drug Administration in the United States or the similar organization in our country, photodynamic therapy was wound to be effective in regards to functional and anatomical outcomes in more than 90% of the CSC patients [5, 11–13]. Several studies have previously evaluated the individual imaging factors, which may affect the treatment outcomes of PDT in CSC, but did not assess each of these different imaging modalities performed in the same cohort [14–17]. In this study we evaluated the associations between the optical coherence tomography (OCT), fluorescein angiography (FA), and ICGA findings and the anatomically PDT response in chronic CSC patients.

## Methods

We reviewed the medical records of the patients with CSC who were admitted to our clinic for the first time between January 2013 and December 2015. The study adhered to the tenets of the Declaration of Helsinki. A written informed consent was obtained from all patients before the PDT. The patients who were diagnosed as CSC, and treatment naïve, and who suffered from the first episode of the disease since at least 6 months, and had a minimum follow-up time of 3 months after the PDT were included in the study. The patients, who had other retinal diseases such as diabetic retinopathy, or retinal vein occlusion, or were diagnosed as recurrent CSC, or CSC secondary to corticosteroid treatment, or underwent any treatment previously were not included.

All patients underwent a standardized examination including measurement of best corrected visual acuity (BCVA) via a projection chart in decimals at 4 m, slit-lamp biomicroscopy, measurement of intraocular pressure via applanation tonometry, and biomicroscopic fundus examination. Fundus photography, fluorescein angiography and ICGA (HRA-2; Heidelberg Engineering, Heidelberg, Germany), and OCT imaging (Spectralis; Heidelberg Engineering, Heidelberg, Germany) were performed. All examinations were repeated at month 1 and 3 monthly, except for FA and ICGA. Optical coherence tomography was used for detecting SRF and measurement of foveal thickness (FT). Foveal thickness, defined as the mean thickness from the internal limiting membrane to the RPE-Bruch’s membrane choriocapillaris complex in a central 1 mm diameter area, was computed using OCT mapping software generated by the device. Central serous chorioretinopathy was diagnosed via FA, ICGA and OCT in the light of previously reported findings [13].

No treatment was given to patients during the first 6 months. Patients were treated with half-fluence PDT after a minimum time of 6 months from the initial signs of the disease, only if they did not show any sign of resolution or were worsened anatomically in regard to FT and presence of subretinal fluid. For the half-fluence PDT procedure, a 689 nm laser system (Carl Zeiss, Dublin, CA, USA) with an indirect lens (Volk Area Centralis, Volk Optical Inc, Mentor, Ohio, USA) was used. All patients underwent a modified fluence PDT with reduced total light energy (25 J/cm^2^) and laser intensity (300 mW/cm^2^) using the standard dose of verteporfin (Visudyne; Novartis AG, Basel, Switzerland) (6 mg/m^2^) and standard time of laser emission (83 s). The PDT spot size was equal to the size of the lesion to cover entire lesion and determined by measuring the greatest linear dimension of the area of choroidal vascular abnormality on ICGA with a device generated software tool.

**Fig. 1:**
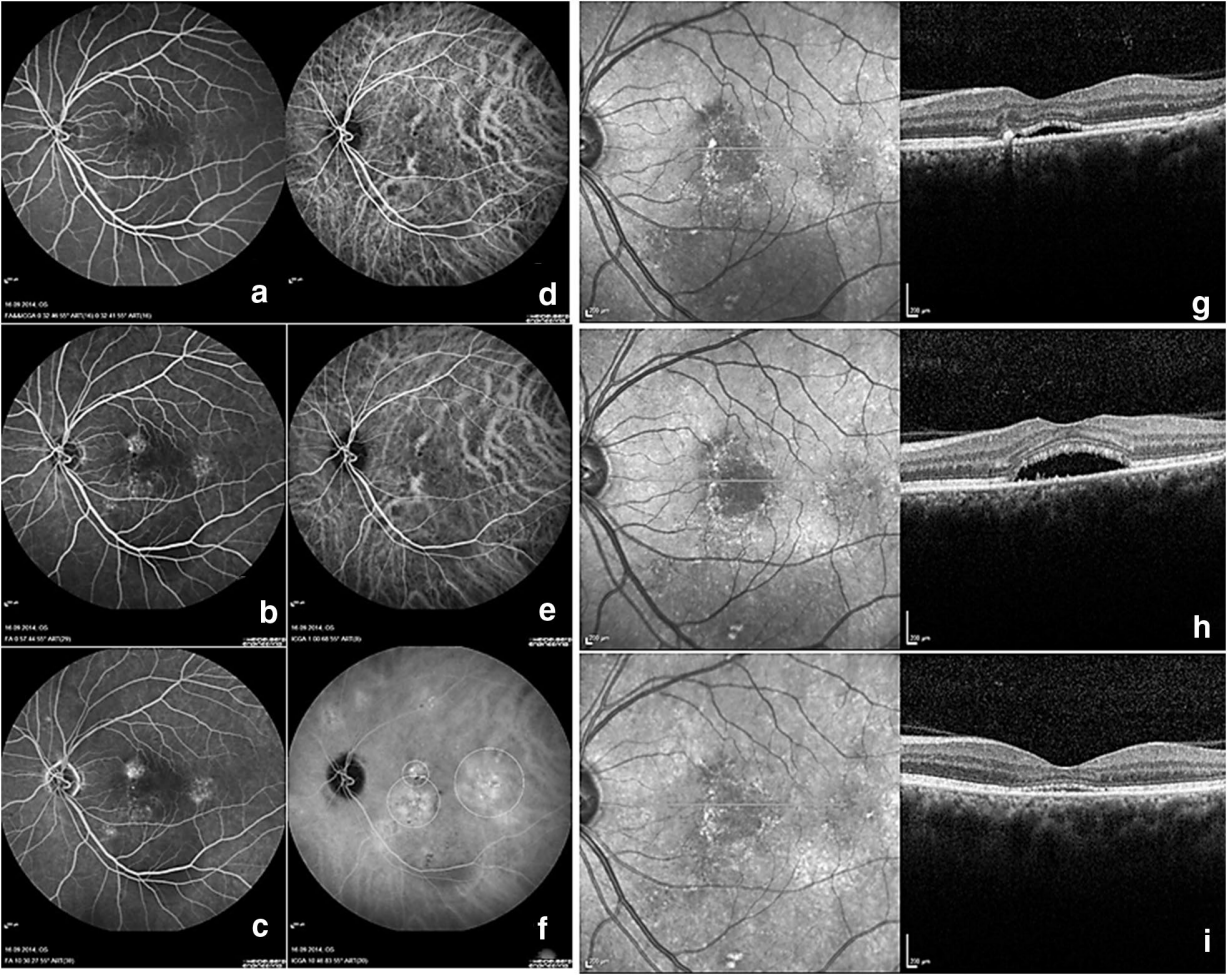
Example of a chronic central serous chorioretinopathy patient who was a good responder to PDT. **a**–**c** Different phases of fluorescein angiography, multifocal diffuse leakage might be detected, **d**–**f** different phases might be seen, dilated choroidal vessels in picture **d** and **e**, and leakage (intense hypercyanescence) from these vessels in picture **f**, **g** pre-treatment optical coherence tomography image, **h** optical coherence tomography image at month 1, the subretinal fluid was increased, **i** subretinal fluid was nearly totally resolved

**Fig. 2:**
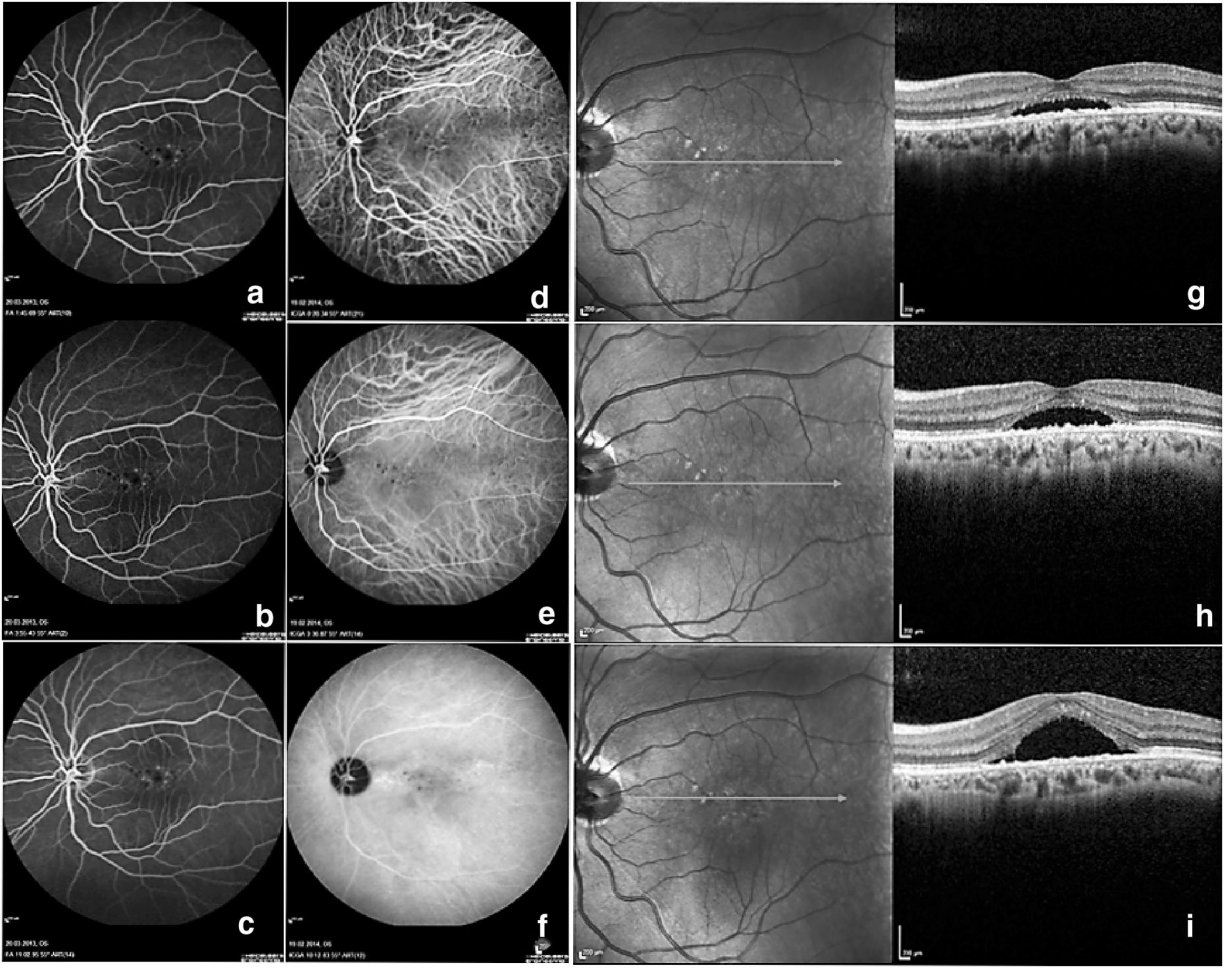
Example of a chronic central serous chorioretinopathy patient who was a poor responder to PDT. **a**–**c** Different phases of fluorescein angiography, diffuse leakage might be detected at the fovea, **d**–**f** different phases might be seen, note the absence of intense hypercyanescence, **g** pre-treatment optical coherence tomography image, **h** optical coherence tomography image at month 1, the subretinal fluid was increased, **i** optical coherence tomography image at month 3, the subretinal fluid was further increased

Main outcome measures of the study were the multimodal imagings findings of the patients obtained via OCT, FA, and ICGA before the PDT. Secondary outcome measures were the visual and anatomical outcomes after the PDT. Optical coherence tomography was evaluated in regards to the presence of retinal pigment epithelium detachment (PED), brush border appearance, intraretinal cystic changes, and hyperreflective dots [18]. Fluorescein angiography was evaluated in regards to the leakage pattern, and the presence of descending track formation. Indocyanine angiography was evaluated in regards to the presence of dilated choroidal vessels at early phases, intense midphase hypercyanescence, and wash out of hypercyanescence at the late phase, and hot plaque formation at the late phase. The success of PDT was evaluated at month 3 and only anatomical success was recorded and the response to PDT was divided into two groups which were good and poor response. Anatomically PDT response based on OCT findings was evaluated and the patients who showed complete resolution of the subretinal fluid, or more than 50% decrease in subretinal fluid by eye examination were accepted as a good responder (Fig. 1). The patients who did not show a significant resolution of subretinal fluid in OCT (<50% by eye examination), or showed an increase in the amount of subretinal fluid in OCT were accepted as poor responders (Fig. 2). The multimodal imaging findings were compared between this two groups. All images were evaluated by two experienced retina specialists (AO, ZTA) according to the defined criteria of the previous studies [13, 14]. The midphase hypercyanescence in ICGA was especially divided into two groups which were intense and weak hypercyanescence groups [13, 14]. There were some confusing cases on which we were not able to make a definite agreement, in such cases, the two specialists jointly adjudicated and arrived at the most probable result.

### Statistical analysis

Visual acuity was converted from decimals to the logarithm of the minimum angle of resolution (LogMAR) for statistical analysis. Categorical variables were presented as numbers and percentages, while numerical variables were expressed as the mean and standard deviation. Categorical variables were analyzed using Chi square test. Wilcoxon’s rank test was used to compare the variables at baseline and after treatment. The Mann–Whitney U-test was used to compare variables between the two groups. The statistical evaluation was performed using SPSS (Version 20.0, SPSS Inc., Chicago, IL, USA). A p value of <0.05 was considered to be statistically significant.

## Results

A total of 101 eyes of 101 patients with the diagnosis of chronic CSC met the inclusion criteria and were included in the study. The mean age of the patients was 51.9 ± 11.1 years (range 26–82 years). Twenty-one patients (20.8%) were female, 80 patients (79.2%) were male. Good responder group was comprised of 76 (75.2%) patients, and poor responder group was comprised of 25 (24.8%) patients. Analysis of the demographic data consisting of sex, age, and symptom duration revealed no significant differences between the two groups (p > 0.05 for all) (Table 1).

**Table 1:** General characteristics of chronic central serous chorioretinopathy patients who were good and poor responders to photodynamic therapy

	Good responder group	Poor responder group	p value
Age	50.7 ± 10.1 years (range 34–82)	55.2 ± 13.3 years (range 26–73)	0.08*
Gender (M/F)	59/17	21/4	0.4^†^
Mean duration of the disease	17.5 ± 12.7 months (range 7–80 months)	15.7 ± 8.4 months (range 7–41 months)	0.5*
Mean baseline BCVA	0.49 ± 0.28 decimals (range 0.01–1.0)	0.37 ± 0.25 decimals (range 0.03–1.0)	0.06*
Mean baseline FT	329 ± 79 microns (range 197–575 microns)	301 ± 57 microns (range 207–399 microns)	0.1*

*M/F* male/female, *BCVA* best corrected visual acuity, *FT* foveal thickness

* Independent-samples T test

^†^Chi square test

### Visual acuity

In the good responder group, the baseline, month 1, and month 3 mean BCVA was 0.49 ± 0.28 decimals (range 0.01–1.0), 0.51 ± 0.31 decimals (range 0.01–1.0), and 0.59 ± 0.31 (range 0.05–1.0), respectively. The mean BCVA at month 1 was not statistically different from the baseline (p = 0.3); however, the mean BCVA was significantly better at month 3 (p = 0.001). In the poor responder group, the baseline, month 1, and month 3 mean BCVA was 0.37 ± 0.25 decimals (range 0.03–1.0), 0.38 ± 0.25 decimals (range 0.03–1.0), and 0.39 ± 0.24 (range 0.03–1.0), respectively. The mean BCVA at month 1 and month 3 was not statistically different from the baseline (p = 0.7 for month 1, and p = 0.9 for month 3, respectively). The change in BCVA was not statistically different between the two groups either at month 1 and month 3, respectively (p = 0.4 for month 1, and p = 0.2 for month 3, respectively).

### Foveal thickness

**Fig. 3:**
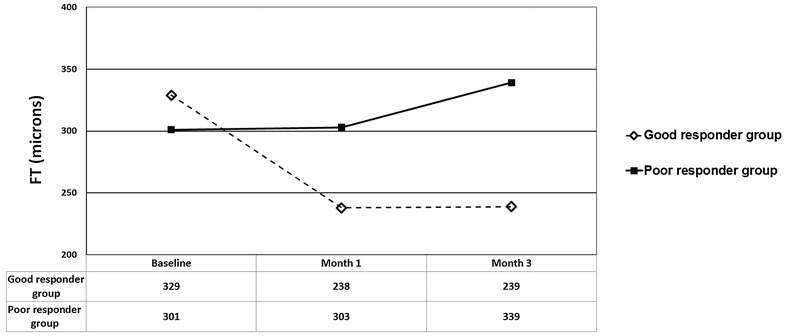
Mean FT changes of chronic central serous chorioretinopathy patients who were good and poor responders to photodynamic therapy. *FT* foveal thickness

The mean FT changes in both groups were shown in Fig. 3. In the good responder group, the baseline, month 1, and month 3 mean FT was 329 ± 79 microns (range 197–575 microns), 238 ± 52 microns (range 141–407 microns), and 239 ± 48 microns (range 155–430 microns), respectively. The mean FT was found to be statistically decreased both at month 1 and 3 from the baseline (p < 0.0001 for month 1, and p < 0.0001 for month 3. In the poor responder group, the baseline, month 1, and month 3 mean FT was 301 ± 57 microns (range 207–399 microns), 303 ± 66 microns (range 191–450 microns), and 339 ± 88 microns (range 224–511 microns), respectively. The mean FT at month 1 was not statistically different from the baseline (p = 0.8); however, the mean FT level at month 3 was higher than baseline (p = 0.007). The change in FT was statistically different between the two groups both at month 1 and month 3, respectively (p < 0.0001 for month 1, and p < 0.0001 for month 3, respectively).

### Multimodal imaging findings

The multimodal imaging findings of the two groups were summarized in Table 2.

**Table 2:** The comparison of multimodal imaging findings between the chronic central serous chorioretinopathy patients who were good and poor responders to photodynamic therapy

	Good responders n, 76	Poor responders n, 25	p value
OCT			
Pigment epithelial detachment	76% (58 eyes)	72% (18 eyes)	0.6*
Brush border appearance	82% (63 eyes)	84% (21 eyes)	0.8*
Intraretinal cycts	82% (63 eyes)	76% (19 eyes)	0.4*
Hyperreflective dots	98% (75 eyes)	100% (25 eyes)	0.5*
FA			
Descending track formation	22% (17 eyes)	32% (8 eyes)	0.3*
FA Leakage pattern (focal/multifocal/diffuse)	18/19/39	3/4/18	0.07*
ICGA			
Midphase hypercyanescence	85% (65 eyes)	48% (12 eyes)	<0.0001*
Dilated choroidal vessels	97% (74 eyes)	96% (24 eyes)	0.7*
Late wash out in ICGA	42% (32 eyes)	52% (13 eyes)	0.4*
Late hot plaque	11% (9 eyes)	4% (1 eye)	0.2*

The significant results were highlighted in italic numbers

*OCT* optical coherence tomography, *FA* fluorescein angiography, *ICGA* indocyanine green angiography

* Chi square test

The percentage of the presence of PED, brush border appearance, intraretinal cysts and hyperreflective dots in OCT were similar between the two groups (p = 0.6, p = 0.8, p = 0.4, p = 0.5, respectively). The distribution of leakage pattern and the percentage of descending track formation in FA were similar between the two groups (p = 0.07, and p = 0.3, respectively). The percentage of the presence of dilated choroidal vessels at early and midphase, late-phase wash out of hypercyanescence, and late phase hot plaque formation in ICGA were similar between the two groups (p = 0.7, p = 0.4, p = 0.2, respectively). The only significantly different imaging finding between the two groups was the presence of intense midphase hypercyanescence (p < 0.0001). Intense midphase hypercyanescence (Fig. 1d–f) was detected in 65 of the 76 patients (85.5%) in the good responder group. In contrast it was detected in only 12 out of the 25 patients (48.0%) in the poor responder group.

No serious complications were detected secondary to PDT in any of the patients.

## Discussion

Photodynamic therapy which was first introduced for the treatment of nAMD is no longer considered as an effective therapeutic option in this indication [8]. Previous studies have shown that PDT caused choroidal atrophy and subsequent vision loss [12, 13]. However, this side effect was found to be useful in PCV and CSC in which the thickening of the choroid was detected [5, 10]. Then several authors reported successful outcomes in CSC patients with PDT [5, 11–17]. In our study, we evaluated several multimodal imaging factors in chronic CSC patients in regard to PDT response. None of the OCT and FA findings were found to be associated with the treatment success of PDT in CSC patients. Only the presence of intense midphase hypercyanescence was found to be associated with a better treatment result. Indeed, this finding was not surprising considering the pathogenesis of CSC [13–17]. The intense hypercyanescence at the midphase ICGA is usually secondary to engorged leaking choroidal vessels, and PDT causes a decrease in the leakage from and thinning of the choroid [3, 5, 13]. Our results support all of these mechanism. We argue that these patients might naturally well respond to PDT as being presented in our study.

In a prospective study by Lim et al. [14], the efficacy of half-fluence PDT on choroidal hyperpermeability in chronic CSC patients were evaluated. The patients were divided into two subgroups as having intense and weak hypercyanescence in the midphase of ICGA. The baseline characteristics of the patients such as mean age, gender distribution, mean baseline BCVA, and FT were similar to our patients. The authors prospectively evaluated the intense and weak hypercyanescence groups in regard to visual and anatomical outcomes, and recurrence. They reported no statistical significance between the two groups in any of the evaluated variables. Inoue et al. [15], also evaluated the association between the efficacy of PDT and ICGA findings of CSC. They evaluated 32 eyes of 27 patients which suffered from CSC since at least 6 months. They divided the patients into 3 subgroups as follows; intense hypercyanescence (23 eyes), intermediate hypercyanescence (6 eyes), and no hypercyanescence (3 eyes). They reported that CSC was resolved in all of the patients who were in intense and intermediate hypercyanescence groups, and none of the three patients in no hypercyanescence group. In addition mean BCVA change was found to be increased in intense and intermediate hypercyanescence groups, and decreased in no hypercyanescence group. In another prognostic study by Moon et al. [17], the prognostic factors related to PDT for CSC were evaluated. They reported that PDT was effective in regard to anatomic and functional outcomes in CSC patients. In addition they revealed that visual improvement might be limited in patients with prolonged disease duration, baseline RPE atrophy, foveal ellipsoid zone disintegrity, and progression of RPE atrophy after PDT. Fujita et al. assessed the outcomes of half-dose verteporfin PDT for chronic CSC patients [19]. The study included 204 eyes of 204 patients and they reported that 89.2% of the patients showed complete resolution of CSC. Also they stated that the patients who did not show complete resolution at month 12 were more likely to have an intermediate hypercyanescence on ICGA and a poorer BCVA before PDT. Our poor responder also had a poorer baseline BCVA than the good responders, but this difference was not statistically significant.

The most important limitation of our study was its retrospective design. Also the good responder group consisted of patients who showed total resolution and subtotal resolution of the CSC. The follow-up period after PDT was relatively short; however, most of the CSC patients show their treatment outcomes during this period [5]. We could not obtain any data about choroidal thickness. Although mean baseline BCVA levels of the two groups did not show statistically significance, the p value was very close to significance (p = 0.06) and the mean baseline BCVA was arithmetically lower in poor responder group which might be created a bias for the study design. In addition we evaluated the posttreatment OCT images by eye examination which was a qualitative analysis and did not use the tracking system of the OCT device which obtains the images from the same point during the follow-up of the patients. The strength of our study was that we used three different imaging techniques and tried to evaluate 10 different findings. And most importantly, our study consisted of a quite good number of patients.

## Conclusions

In conclusion, the OCT or FA findings evaluated in this study were not associated with anatomically PDT response in chronic CSC patients. The midphase intense hypercyanescence on ICGA was the only finding which seemed to have an effect on the anatomically PDT response. Therefore, while informing the CSC patients before undergoing PDT, we may use this parameter as a prognostic factor.

## Abbreviations

CSC: chronic central serous chorioretinopathy
PDT: photodynamic therapy
OCT: optical coherence tomography
FA: fluorescein angiography
ICGA: indocyanine green angiography
RPE: retinal pigment epithelium
EDI-OCT: enhanced depth imaging optical coherence tomography
SRF: subretinal fluid
nAMD: neovascular age-related macular degeneration
PCV: polypoidal choroidal vasculopathy
PPE: pachychoroid pigment epitheliopathies
BCVA: best corrected visual acuity
ETDRS: Early Treatment Diabetic Retinopathy Study
PED: retinal pigment epithelium detachment
LogMAR: logarithm of the minimum angle of resolution
FT: foveal thickness

## References

[1] Liu B, Deng T, Zhang J (2016). Risk factors for central serous chorioretinopathy: a systematic review and meta-analysis. Retina.

[2] Fine HF, Ober MD, Hariprasad SM (2014). Current concepts in managing central serous chorioretinopathy. Ophthalmic Surg Lasers Imaging Retina.

[3] Agrawal R, Chhablani J, Tan KA, Shah S, Sarvaiya C, Banker A (2016). Choroidal vascularity index in central serous chorioretinopathy. Retina.

[4] Yung M, Klufas MA, Sarraf D (2016). Clinical applications of fundus autofluorescence in retinal disease. Int J Retina Vitreous.

[5] Alkin Z, Ozkaya A, Agca A (2014). Early visual and morphologic changes after half-fluence photodynamic therapy in chronic central serous chorioretinopathy. J Ocul Pharmacol Ther.

[6] Kim HC, Cho WB, Chung H (2012). Morphologic changes in acute central serous chorioretinopathy using spectral domain optical coherence tomography. Korean J Ophthalmol.

[7] Daruich A, Matet A, Dirani A (2015). Central serous chorioretinopathy: recent findings and new physiopathology hypothesis. Prog Retin Eye Res.

[8] Newman DK (2016). Photodynamic therapy: current role in the treatment of chorioretinal conditions. Eye (Lond)..

[9] Warrow DJ, Hoang QV, Freund KB (2013). Pachychoroid pigment epitheliopathy. Retina.

[10] Quaranta M, Mauget-Faÿsse M, Coscas G (2002). Exudative idiopathic polypoidal choroidal vasculopathy and photodynamic therapy with verteporfin. Am J Ophthalmol.

[11] Cheng CK, Chang CK, Peng CH (2017). Comparison of photodynamic therapy using half-dose of verteporfin of half-fluence of laser light for the treatment of chronic central serous chorioretinopathy. Retina.

[12] Ozkaya A, Alkin Z, Ozveren M, Yazici AT, Taskapili M (2016). The time of resolution and the rate of recurrence in acute central serous chorioretinopathy following spontaneous resolution and low-fluence photodynamic therapy: a case control study. Eye (Lond.).

[13] Liegl R, Ulbig MW (2014). Central serous chorioretinopathy. Ophthalmologica.

[14] Lim SH, Chang W, Sagong M (2013). Efficacy of half-fluence photodynamic therapy depending on the degree of choroidal hyperpermeability in chronic central serous chorioretinopathy. Eye (Lond.).

[15] Inoue R, Sawa M, Tsujkawa M, Gomi F (2010). Association between the efficacy of photodynamic therapy and indocyanine green angiography findings for central serous chorioretinopathy. Am J Ophthalmol.

[16] Schliesser JA, Gallimore G, Kunjukunju N, Sabates NR, Koulen P, Sabates FN (2014). Clinical application of optical coherence tomography in combination with functional diagnostics: advantages and limitations for diagnosis and assessment of therapy outcome in central serous chorioretinopathy. Clin Ophthalmol.

[17] Moon JW, Yu HG, Kim TW, Kim HC, Chung H (2009). Prognostic factors related to photodynamic therapy for central serous chorioretinopathy. Graefes Arch Clin Exp Ophthalmol.

[18] Teke MY, Elgin U, Nalcaciolglu-Yuksekkaya P, Sen E, Ozdal P, Ozturk F (2014). Comparison of autofluorescence and optical coherence tomography findings in acute and chronic central serous chorioretinopathy. Int J Ophthalmol.

[19] Fujita K, Imamura Y, Shinoda K (2015). One-year outcomes with half-dose verteporfin photodynamic therapy for chronic central serous chorioretinopathy. Ophthalmology.

